# Small left ventricular size as a predictor for incident type 2 diabetes: insights from the UK biobank cardiovascular magnetic resonance substudy

**DOI:** 10.1186/s13098-025-01939-7

**Published:** 2025-10-03

**Authors:** Gaifeng Hu, Xiaodong Peng, Liu He, Zixu Zhao, Caihua Sang, Jianzeng Dong, Changsheng Ma

**Affiliations:** Department of Cardiology, Beijing AnZhen Hospital, National Clinical Research Center for Cardiovascular Diseases, Capital Medical University, Office of Beijing Cardiovascular Diseases Prevention, No.2 Anzhen Rd, Chaoyang District, Beijing, 100029 China

**Keywords:** Type 2 diabetes, Cardiovascular, Magnetic resonance imaging, Predictor

## Abstract

**Background:**

Left ventricular (LV) concentric hypertrophy is common in diabetes patients, presenting as a relatively small LV size. However, studies have shown that a small LV size can also occur in prediabetic conditions without ventricular hypertrophy. We used data from the UK Biobank Cardiovascular Magnetic Resonance Substudy to assess whether a small LV size independently predicts incident type 2 diabetes.

**Methods and results:**

Small LV size was defined using indexed left ventricular end-diastolic volume (iLVEDV) values (< 56 mL/m² for females and < 57 mL/m² for males). The risk of small LV size for incident type 2 diabetes was assessed using adjusted Cox proportional hazards models. The non-linear relationship between iLVEDV and diabetes risk was evaluated using restricted cubic splines. This study included 35,422 participants, with an average age of 64 years, of whom 53.2% were females. Among the 35,422 participants, 947 (2.7%) had small LV size. During a median follow-up of 698 days, 304 cases of incident type 2 diabetes were recorded. Those with small LV size showed a significant association with increased risk of incident type 2 diabetes (adjusted hazard ratio [HR], 2.36; 95% CI, 1.56–3.57). Subgroup analysis consistently supported this relationship across age, sex, hypertension, obesity, and genetic risk for type 2 diabetes. An L-shaped relationship between iLVEDV and diabetes risk was also observed.

**Conclusions:**

Small LV size is an independent predictor of incident type 2 diabetes, with a smaller LV size correlating with a higher risk of developing the condition, warranting further investigation.

**Graphical Abstract:**

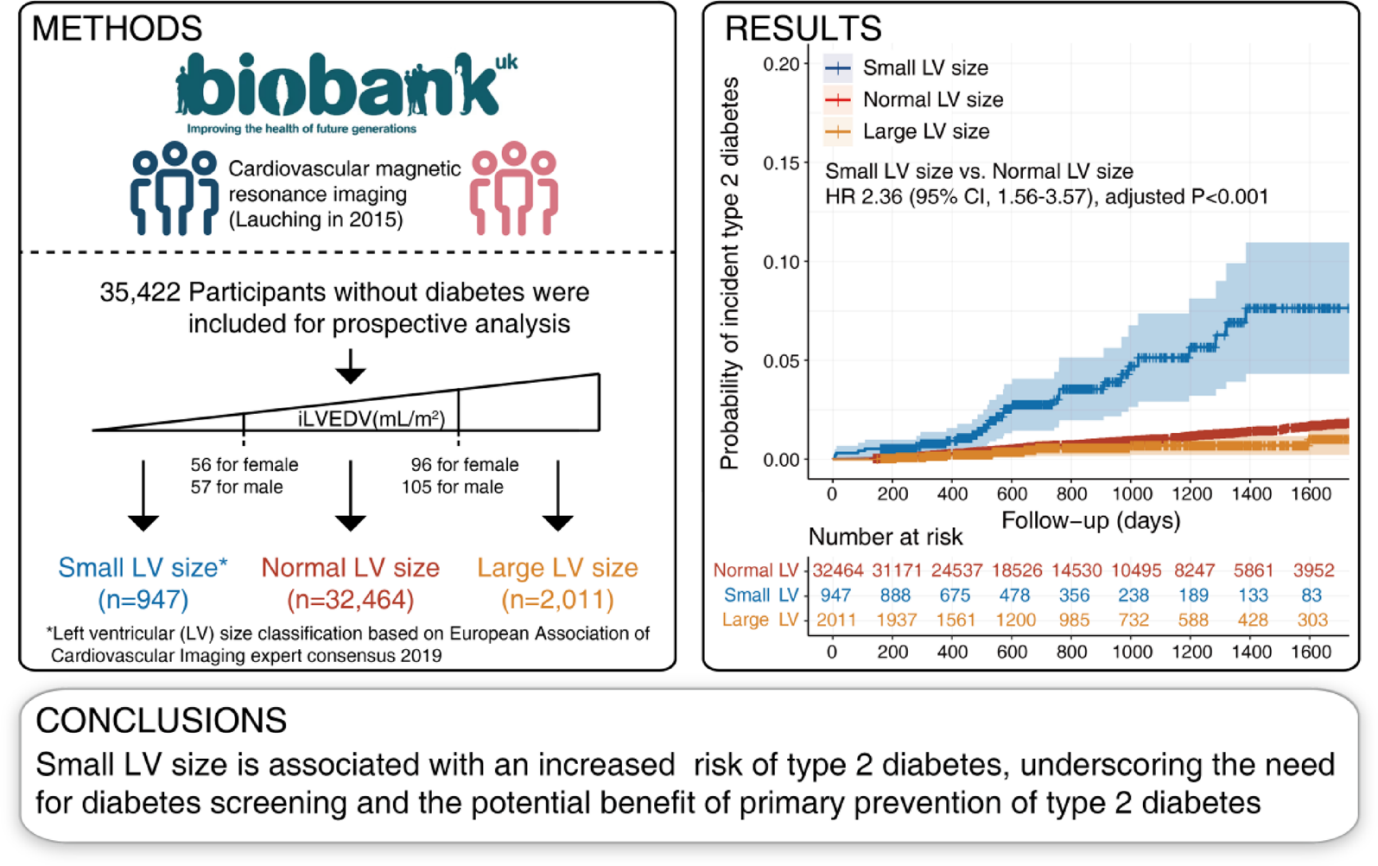

**Supplementary Information:**

The online version contains supplementary material available at 10.1186/s13098-025-01939-7.

## Highlights

- Small left ventricular size is observed in 2.7% of the study sample without diabetes.
- Small left ventricular size is an independent predictor of incident type 2 diabetes, with a smaller LV size correlating with a higher risk of developing the condition.
- Individuals with a small left ventricular size may be at a high risk for developing type 2 diabetes and should undergo regular diabetes screening.

**Supplementary Information:**

The online version contains supplementary material available at 10.1186/s13098-025-01939-7.

## Introduction

Type 2 diabetes is the leading risk factor for mortality and reduced quality of life globally, irrespective of economic status [1, 2]. Based on statistical data from the International Diabetes Federation (IDF), approximately 10.5% adults (over half a billion) were affected by type 2 diabetes in 2021, with a continuing increase projected to reach 12.2% of population by 2045 [3]. It is of practical importance to identify the vulnerable individuals for type 2 diabetes and intervene early to reduce their risk of developing this condition [4], thereby mitigating the healthcare burden associated with its complications [5].

Structural remodeling of the left ventricle (LV) is a key characteristic of type 2 diabetes [6], and recent findings suggest that LV structural abnormalities, manifesting as small LV size, may precede the clinical diagnosis of type 2 diabetes [7–9]. A recently published study revealed that small LV size is associated with adverse outcomes in individuals with cardiovascular disease [10]. Myocardial involvement related to type 2 diabetes typically manifests as decreased LV size due to increased LV mass. However, a community-based study revealed that small LV size without LV hypertrophy was associated with pre-diabetic condition [11]. Despite the hypothesis has been put forward to draw attention on the linkage of small LV size and diabetes, evidence from large-scale and high-quality data is still warranted.

Frailty index, an aggregate measure of health deficits, has emerged as a relevant marker of metabolic vulnerability and risk for adverse outcomes including diabetes. Thus, its inclusion in cardiometabolic research warrants explicit justification [12].

Therefore, we aimed to investigate the association between small LV size and incident type 2 diabetes using CMR imaging data from the UK Biobank, and to explore the role of frailty and other covariates in this association.

## Materials and methods

All data used in this work can be acquired from the UK Biobank (https://www.ukbiobank.ac.uk/), subject to obtaining official permission from UK Biobank.

### Study design and data source

The UKB study is a prospective, longitudinal research that enrolls participants primarily from England (89%), with additional representation from Scotland (7%) and Wales (4%). It constitutes a substantial biomedical repository containing anonymized health data sourced from half a million individuals aged 40 to 69 who have committed to long-term follow-up. The collection of individual data occurred upon admission, and their medical records are seamlessly linked through national medical systems using their National Health Service (NHS) number (for England or Wales) or Community Health Index (CHI) number (for Scotland). The imaging assessment project within the UKB was initiated in 2015, randomly selecting 20% of participants from the original cohort. A comprehensive rationale for CMR imaging in participants of UKB is publicly available [13].

Overall, nearly 40,000 participants completed the CMR assessment for LV size and function before the onset of COVID-19 outbreak in 2020 across four imaging assessment centers (Cheadle, Reading, Newcastle and Bristol) and their data are currently accessible. The UKB dataset had obtained written informed consent from all participants and ethical approval from the National Research Ethics Service Committee (reference number 11/NW/0382), adhering to the Declaration of Helsinki principles. This study utilized data extracted from UKB under an approved project (application number 103736). Other Clinical trial number: not applicable.

### Cardiovascular magnetic resonance procedures

CMR imaging was performed according to the standardized UK Biobank protocol [14], utilizing a 1.5 Tesla scanner (MAGNETOM Aera, Siemens Healthcare). Image acquisition included balanced steady-state free precession (bSSFP) cine sequences for ventricular volumetry and function assessment, with standardized horizontal/vertical long-axis and short-axis stacks covering the entire left ventricle. Quality control was implemented through real-time assessment by trained radiographers and post-acquisition review by senior staff, as detailed in the UK Biobank imaging protocol [14]. To ensure consistent image quality throughout the study, the Cardiac Dot Engine (Siemens Healthcare, Erlangen, Germany) was employed to aid in image acquisition.

CMR acquisitions conducted by the UK Biobank encompass a range of scans, including piloting and partial coverage of the chest and abdomen in sagittal, transverse, and coronal views. For assessing cardiac function, various cine sequences are employed, including horizontal and vertical long-axis views, sagittal and coronal LV outflow tract cine images, and a comprehensive short-axis stack covering the LV, using balanced steady-state free precession.

All radiographers undergo standardized training, ensuring adherence to established scanning protocols. Real-time assessment of scan completeness is conducted during acquisition, with subsequent evaluation of image quality and artifacts performed by a senior radiographer post-acquisition.

### Left ventricular size classification

The classification of LV size relies on the expert consensus of the European Association of Cardiovascular Imaging, with indexed left ventricular end-diastolic volume (iLVEDV) serving as the metric [15]. The formula for iLVEDV involves dividing the left ventricular end-diastolic volume (LVEDV) by the body surface area (BSA). BSA was derived using the Mosteller formula: BSA (m²) = √([height (cm) × weight (kg)]/3600) [16]. The established reference range for iLVEDV is 56–96 mL/m^2^ for females and 57–105 mL/m^2^ for males. Accordingly, individuals with iLVEDV < 56 mL/m^2^ for females and < 57 mL/m^2^ for males are categorized as having a small LV size, while those exceeding the upper limit of the reference range are considered to have a large LV size.

### Covariates definition and incident type 2 diabetes ascertainment

The covariates used to characterize participant traits and document variable definitions with corresponding UK Biobank data-field codes are listed in Table S1. The specific covariates included as confounders and adjusted for in the Cox regression models are explicitly listed in Table 1. This encompasses socio-demographic data, lifestyle factors, family history of diabetes, physical measurements, medical conditions, and polygenic risk scores (PRS) for type 2 diabetes, all of which were extracted to examine the association between small LV size and type 2 diabetes. Those potential confounders were selected a priori based on established associations with both LV structure and type 2 diabetes, and included socio-demographic characteristics (age, sex, British descent/ethnicity, Townsend deprivation index, and assessment center) [13, 17–19], lifestyle factors (smoking status and alcohol intake frequency) [20, 21], family history of diabetes [22], metabolic indicators (body mass index, waist circumference, hypertension) [23, 24], and CMR-derived cardiac structure and function parameters (LV mass, LV global longitudinal strain, LV ejection fraction, and LA ejection fraction) [6, 25, 26]. Polygenic risk scores for type 2 diabetes were included in the fully adjusted model to account for genetic susceptibility [27, 28]. The number of covariates was determined according to the “events per variable” (EPV) principle, using the total number of events across all exposure groups, as recommended in methodological guidelines [29]. Furthermore, the correlation between small LV size and frailty was also assessed. The variables utilized for calculating the frailty index (FI) in UKB have been previously reported [30], and are outlined in Table S2 in detail.

**Table 1:** Association of left ventricle size and incident type 2 diabetes using Cox proportional hazard model

	Events No.	Participants No.	Hazard ratio (95% CI)		
			Model 1	Model 2	Model 3
Continuous variable					
iLVEDV per 10mL/m^2^ decrease	304	35 422	1.28(1.16–1.41) *P* < 0.001	1.38 (1.22–1.55) *P* < 0.001	1.36 (1.21–1.54) *P* < 0.001
Categorical variable*					
Normal LV size	264	32 464	Reference	Reference	Reference
Small LV size	30	947	2.52 (1.71–3.71) *P* < 0.001	2.45 (1.62–3.70) *P* < 0.001	2.36 (1.56–3.57) *P* < 0.001
Large LV size	10	2 011	0.76 (0.40–1.43) *P* = 0.396	0.78 (0.40–1.53) *P* = 0.474	0.79 (0.40–1.54) *P* = 0.476

Model 1: age, sex, British descent, Townsend deprivation index (TDI), assessment centers, family history of diabetes, hypertension, body mass index, waist circumference, smoking status and alcohol intake frequency

Model 2: Model 1 plus, left ventricular mass, left ventricular longitudinal strain global, left ventricular ejection fraction and left atrial ejection fraction

Model 3: Model 2 plus polygenic risk scores for type 2 diabetes

*Classification of left ventricle size was based on the European Association of Cardiovascular Imaging expert consensus on cardiovascular magnetic resonance normal values of cardiac chamber size

This study defined incident type 2 diabetes as the endpoint event, which was determined based on International Classification of Diseases-10 (ICD-10) codes extracted from healthcare and death register records (E11.0, E11.1, E11.2, E11.3, E11.4, E11.5, E11.6, E11.7, E11.8, E11.9). Individuals were categorized as having a history of diabetes if their initial diagnosis occurred prior to their attendance at the CMR assessment centers or if they were undergoing diabetes medication at the time of the CMR examination. The diabetes medications were defined as oral agents including metformin, sulfonylureas, and DPP-4 inhibitors. Those with a history of diabetes were excluded from the analysis (*n* = 2 039), as were individuals with a history of cardiomyopathy (*n* = 157).

### Statistical analysis

Continuous variables were expressed as mean ± standard deviation (SD) for normally distributed data or median (interquartile range, IQR) for non-normally distributed data. Categorical variables were presented as counts and percentages. Normality was assessed using the Kolmogorov–Smirnov test and visual inspection of Q–Q plots. Comparisons of normally distributed continuous variables among three groups were performed using one-way analysis of variance (ANOVA) followed by the Least Significant Difference (LSD) post hoc test. Non-normally distributed continuous variables were compared using the Mann–Whitney U test (two groups) or the Kruskal–Wallis test (more than two groups). Categorical variables were compared using the chi-square test or Fisher’s exact test, as appropriate.

The association between small LV size and incident type 2 diabetes was assessed using multi-variable adjusted Cox proportional hazards models. Multicollinearity among model covariates was assessed prior to regression analysis by calculating both variance inflation factors (VIFs) and tolerance statistics using SPSS. Individuals at risk of type 2 diabetes were tracked since the date of their attendance at CMR assessment centers, and were censored until the earliest of the following events: (1) occurrence of the endpoint event (type 2 diabetes), (2) non-type 2 diabetes-related death, (3) loss to follow-up, or (4) the onset of the COVID-19 outbreak in the UK (January 31, 2020). It is noteworthy that the last follow-up time was required to precede the onset of COVID-19, otherwise designated as January 31, 2020. This precaution aimed to mitigate the potential impact of COVID-19 on the recording of endpoint events. A Kaplan-Meier survival curve was constructed to illustrate the time to event for three LV sizes (small, normal and large). The non-linear relationship between LV size (iLVEDV) and the risk of type 2 diabetes (adjusted hazard ratio [HR]) was evaluated using restricted cubic spline (RCS). Multiple imputation using the random forest method was employed to handle missing data in the covariates. This approach accommodates both nonlinear interactions and mixed data types and has been validated in biomedical datasets [31]. The robustness of imputation was evaluated by conducting a complete-case sensitivity analysis.

Subgroup analysis was conducted to assess the effect modification of genetic and other metabolic risk factors on the association between small LV size and type 2 diabetes. The genetic risk for type 2 diabetes was stratified into low (lowest PRS tertile, PRS<−0.647), intermediate (middle PRS tertile, −0.647 ≤ PRS ≤ 0.160), and high (highest PRS tertile, PRS > 0.160) categories based on the standard PRS validated in genome-wide association studies [28]. Furthermore, the interaction effect of age, sex, hypertension, obesity, and family history of diabetes on the association between small LV size and type 2 diabetes has been evaluated. Because small LV size was associated with a higher LV mass‑to‑volume ratio, which reflects concentric remodeling and could potentially mediate the relationship between LV size and diabetes, we conducted a sensitivity analysis with additional adjustment for this parameter to evaluate whether the observed association was independent of LV concentric remodeling [32].

The linear correlation between cardiometabolic indicators (systolic blood pressure, body mass index, waist circumference and heart rate), cardiac structure (LV mass), cardiac function (LV ejection fraction, LV longitudinal strain, LV cardiac output and LV stroke volume) and LV size was estimated using spearman correlation analysis. The non-linear relationship between these parameters and LV size was assessed using generalized additive model (GAM). Furthermore, the association between frailty and small LV was estimated using multi-variable adjusted logistics regression model, and the dose-response relationship between FI and odds ratio of small LV was visualized through fitted RCS. For the analysis related to frailty, participants who had missing values on ≥ 10 variables used for calculating the FI were excluded. Based on the FI, participants were categorized into non-frailty (FI ≤ 0.10), pre-frailty (0.10 < FI ≤ 0.21), and frailty (FI > 0.21) [33].

To validate the robustness of the association between small LV size and type 2 diabetes, several sensitivity analyses were conducted: (1) a competing risk regression model was employed to mitigate potential bias stemming from the presence of competitive event (all-cause death); (2) age was treated as the time-scale in Cox proportional hazards models to re-evaluate the results in this prospective, observational study(14); (3) participants with recorded glycated hemoglobin (HbA1c) levels > 5.7%, indicative of pre-diabetic status, were additionally excluded (*n* = 3 620); (4) participants with missing data on covariates were excluded (*n* = 8 171); (5) additional adjustments were made for LV mass to volume ratio in the Cox proportional hazards models. (6)Although events per variable is defined with respect to the total number of events in the model [29], sparse events in the exposure category may still induce sparse-data bias and wide confidence intervals [34], we therefore applied Firth penalized Cox regression and reduced-variable sensitivity analyses [35].

The examination was carried out utilizing SPSS (IBM SPSS Statistics for Windows, version 26.0, IBM Corp., Armonk, NY) and R version 4.3.1, developed by The R Foundation for Statistical Computing in Vienna, Austria. The “mice” R package was employed for multiple imputation, and the “rms” R package was utilized for RCS evaluation and visualization. The “cmprsk” R package was applied for the analysis of competing risk model, and the “mgcv” R package was utilized for conducting the GAM analysis. A significance threshold of *P* < 0.05 was employed for two-sided testing.

## Results

### Baseline characteristics

**Fig. 1:**
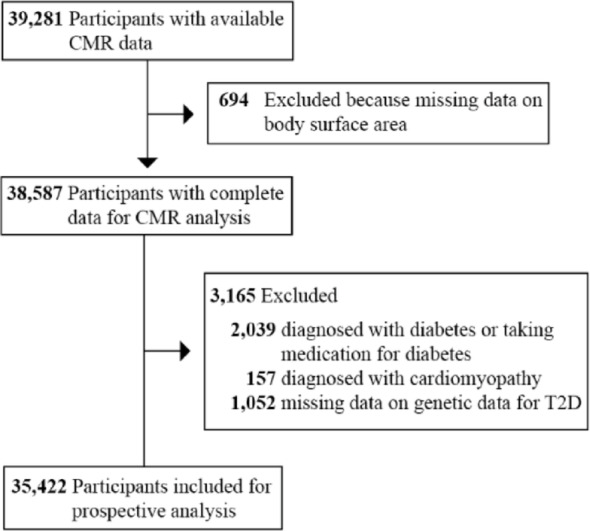
Flowchart outlining the process for selecting the study sampleCMR, cardiac magnetic resonance; CVD, cardiovascular diseases

A total of 35,422 participants, with a median follow-up time of 698 days, were included for analysis (Fig. 1). The Kolmogorov–Smirnov test indicated that only PRS for T2D was normally distributed (*p* = 0.145), while all other continuous variables significantly deviated from normality (*p* < 0.001) (Table S8). Among them, 947 (2.67%) were classified as having a small LV size. Individuals with a small LV size were older and had higher levels of body mass index (BMI), waist circumference, and heart rate, but lower birth weight compared to those with normal and large LV sizes (Table 2). A higher prevalence of hypertension, obesity, and female sex was observed among participants with a small LV size. Additionally, these individuals exhibited a higher FI and PRS for type 2 diabetes. In terms of cardiac structure and function, those with a small LV size had lower levels of LV mass, LV stroke volume, and LV cardiac output, but higher LV ejection fraction (LVEF) and LV mass to volume ratio.

**Table 2:** Baseline characteristics

**Variables**		Classification according to LV size			
	**Whole population**	**Normal**	**Small**	**Large**	P-value*
Total. No.	35 422	32 464	947	2 011	
Age, years	62(12)	62.00 (12)	67.00 (9)^a, b^	60.00 (12)	< 0.001
Female, n (%)	18 845 (53.2)	17 524 (54.0)	657 (69.4)^a, b^	664 (33.0)	< 0.001
British descent, n (%)	32 498 (91.7)	29 782 (91.7)	864 (91.2)	1 852 (92.1)	0.723
Smoking status, n (%)					< 0.001
Never	22 350 (63.1)	20 589 (63.4)	521 (55.0)^a, b^	1 240 (61.7)	
Previous	1 215 (3.4)	1 104 (3.4)	38 (4.0)	73 (3.6)	
Current	11 857 (33.5)	10 771 (33.2)	388 (41.0)^a, b^	698 (34.7)	
Alcohol frequency, n (%)					< 0.001
Never or occasionally	5 756 (16.2)	5 248 (16.2)	247 (26.1)^a, b^	261 (13.0)	
Intake regularly^#^	23 655 (66.8)	21 730 (66.9)	547 (57.8)^a, b^	1 378 (68.5)	
Daily or almost daily	6 011 (17.0)	5 486 (16.9)	153 (16.2)	372 (18.5)	
TDI	−2.65 (3.30)	−2.67 (3.30)	−2.41 (3.84)^b^	−2.56 (3.26)	0.04
SBP, mmHg	138 (26)	138 (26)	141 (24)^a^	140 (27)	< 0.001
DBP, mmHg	78 (14)	78 (14)	81(15)^a, b^	78 (15)	< 0.001
Heart rate, bpm	61 (13)	62.00 (12)	73.00 (15)^a, b^	53.00 (11)	< 0.001
BMI, kg/m^2^	25.66 (5.27)	25.68 (5.27)	27.33 (5.92)^a, b^	24.73 (4.66)	< 0.001
Waist circumference, cm	86.00 (17.60)	86.00 (17.6)	90.00 (19.0)^a, b^	84.00 (16.0)	< 0.001
Birth weight, kg	3.37 (0.68)	3.37 (0.68)	3.18 (0.74)^a, b^	3.40 (0.65)	< 0.001
Hypertension, n (%)	9 600 (27.1)	8 762 (26.9)	298 (31.5)^a^	576 (28.6)	0.002
Antihypertensive medication, n (%)	7 336 (20.7)	6 649 (20.5)	251 (26.5)^a, b^	436 (21.7)	< 0.001
Obesity^##^, n (%)	5 938 (16.8)	5 464 (16.8)	259 (27.3)^a, b^	215 (10.7)	< 0.001
Dyslipidemia, n (%)	6 976 (19.7)	6 372 (19.6)	278 (29.4)	326 (16.2)	< 0.001
Family history of diabetes, n (%)	5 618 (15.9)	5 165 (15.9)	167 (17.6)^b^	286 (14.2)	0.042
Fragility index	0.12 (0.10)	0.125 (0.103)	0.138 (0.108)^a, b^	0.113 (0.097)	< 0.001
PRS for type 2 diabetes	−0.24 ± 0.95	−0.24 ± 0.94	−0.11 ± 0.98^a, b^	−0.30 ± 0.96	< 0.001
iLVEDV, mL/m^2^	77.78 (17.17)	77.28 (15.43)	53.24 (4.39)^a, b^	108.28 (11.37)	< 0.001
iLVESV, mL/m^2^	30.91 (10.06)	30.60 (9.10)	19.83 (4.17)^a, b^	46.43 (11.58)	< 0.001
LV stroke volume, mL	84.81 (25.16)	84.18 (23.30)	57.64 (11.62)^a, b^	116.25 (27.93)	< 0.001
LV cardiac output, L/min	5.23 (1.60)	5.20 (1.55)	4.21 (1.28)^a, b^	6.20 (1.80)	< 0.001
Indexed LV myocardial mass, g/m^2^	43.74 (11.22)	43.39 (10.45)	35.33 (7.07)^a, b^	57.07 (12.92)	< 0.001
LV longitudinal strain global, %	−18.53 (3.41)	−18.57 (3.40)	−18.06 (3.31)^a^	−18.11 (3.57)	< 0.001
LVEF, %	59.91 (7.69)	60.01 (7.67)	62.20 (6.93)^a, b^	57.23 (8.32)	< 0.001
LA maximum volume, mL	69.66 (27.82)	69.06 (26.54)	51.53 (20.86)^a, b^	93.42 (33.38)	< 0.001
LA minimum volume, mL	26.76 (15.04)	26.45 (14.51)	19.15 (12.49)^a, b^	37.43 (19.24)	< 0.001
LAEF, %	61.38 (9.74)	61.51 (9.67)	62.26 (12.00)^b^	59.19 (9.69)	< 0.001

*Comparison among three LV size groups using ANOVA (normal distribution expressed as mean ± SD) or Kruskal–Wallis H (non-normally distributed variables expressed as median (IQR)) or chi-square test

^a^Statistically significant differences (*P* < 0.05) were found between small and normal LV size

^b^Statistically significant differences (*P* < 0.05) were found between small and large LV size

^#^indicates alcohol intake one to three times a month, once or twice a week, and three or four times a week

^##^Obesity include body mass index ≥ 30 kg/m^2^ and abdominal obesity (waist circumference ≥ 105 cm for female and ≥ 110 cm for male)

BMI, body mass index; DBP, diastolic blood pressure; iLVEDV, indexed left ventricular end diastolic volume; iLVESV, indexed left ventricular end systolic volume; LAEF, left atrial ejection fraction; LV, left ventricle; LVEF, left ventricular ejection fraction; PRS, polygenic risk scores; SBP, systolic blood pressure; TDI, Townsend deprivation index; type 2 diabetes, type 2 diabetes

### Association between small left ventricular size and incident type 2 diabetes

**Fig. 2:**
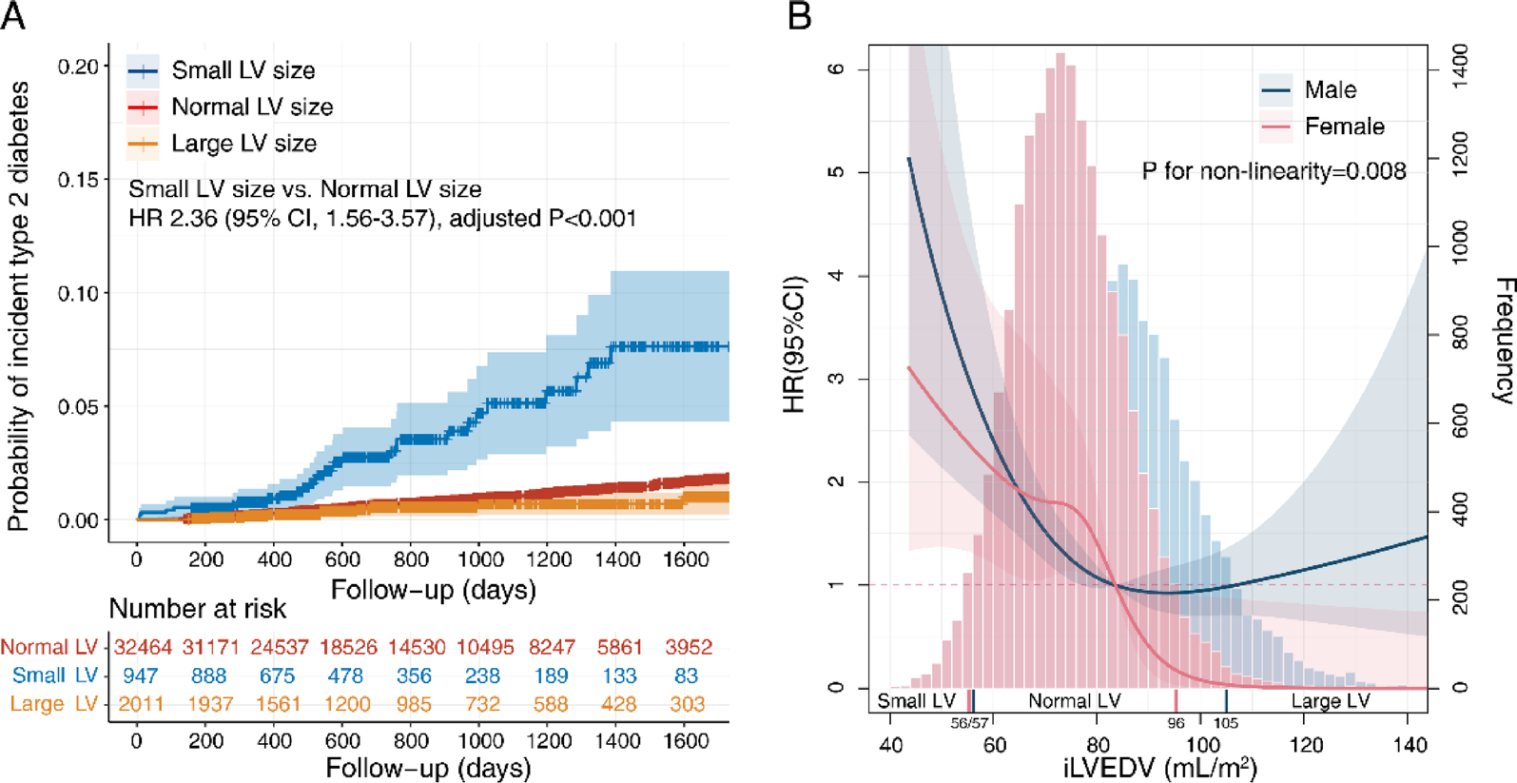
Kaplan-Meier curves and non-linear relationship between left ventricular size and incident type 2 diabetes. Cox adjusted for age, sex, British descent, Townsend deprivation index (TDI), assessment centers, family history of diabetes, hypertension, body mass index, waist circumference, smoking status, alcohol intake frequency, left ventricular longitudinal strain global, left ventricular ejection fraction, left atrial ejection fraction and polygenic risk scores for type 2 diabetes. CI, confidence interval; HR, hazard ratio

During the follow-up period, 264, 30, and 10 cases of incident type 2 diabetes events were recorded among individuals with normal, small, and large LV sizes, respectively. When comparing to the normal LV size group as the reference, the risk of developing type 2 diabetes significantly increased in the small LV size group (adjusted HR, 2.36; 95% CI, 1.56–3.57) (Fig. 2A), whereas no significant difference was observed in the large LV size group (adjusted HR, 0.79; 95% CI, 0.40–1.54, *P* = 0.476). Furthermore, after adjusting for various covariates, every 10 mL/m^2^ decrease in iLVEDV was associated with a 36% increase in the risk of type 2 diabetes (Table 1). Notably, all model covariates demonstrated tolerance values greater than 0.2 and VIFs less than 5, indicating that multicollinearity was not a concern (see Supplementary Table S7). Additionally, a non-linear relationship was identified between iLVEDV and the risk of type 2 diabetes, whereby a higher risk of type 2 diabetes was correlated with a smaller iLVEDV, but such correlation became insignificant with the increase in iLVEDV (Fig. 2B).

### Subgroup analysis stratified by genetic risk and covariates

**Fig. 3:**
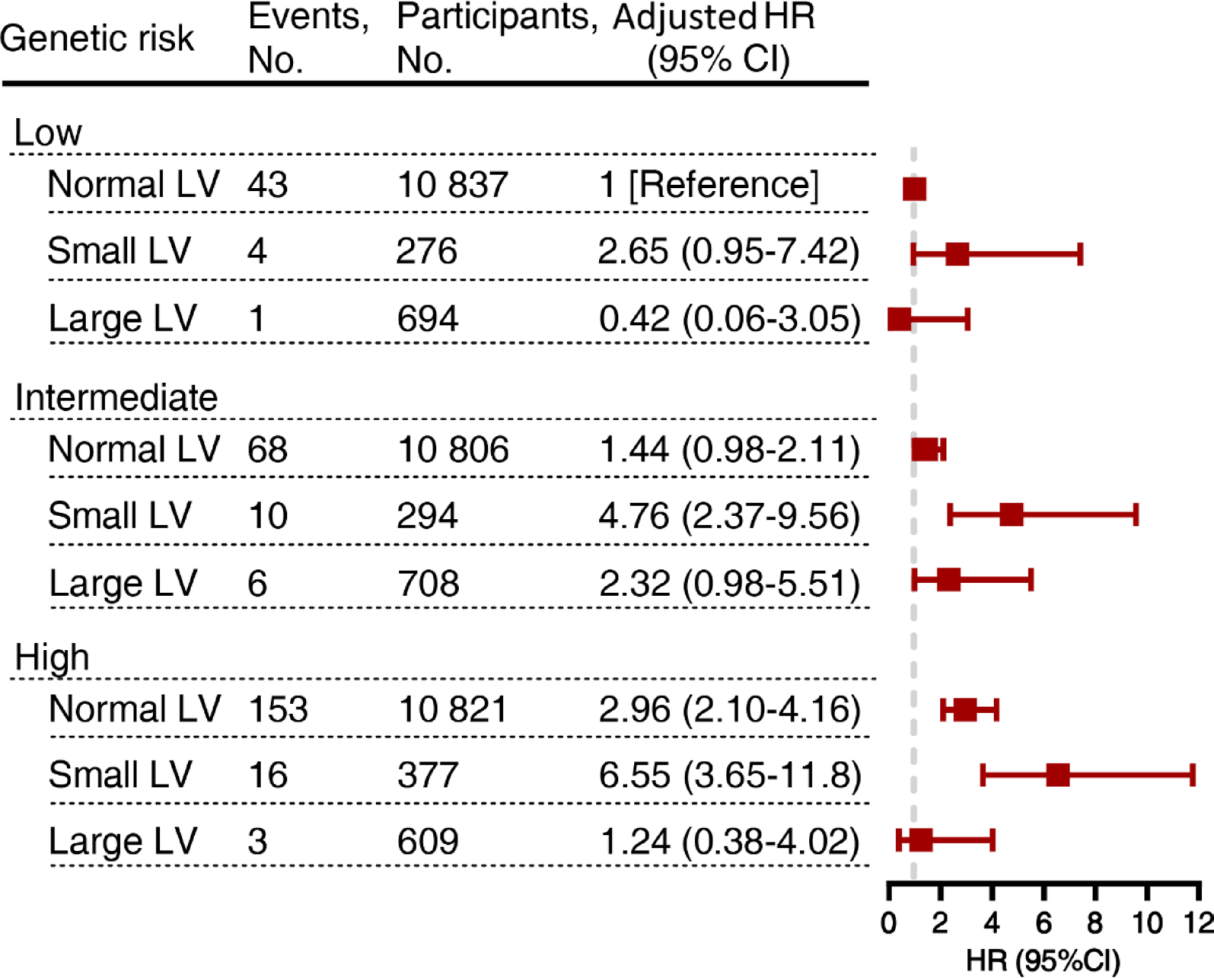
Association of left ventricle size and incident type 2 diabetes according to polygenic risk stratification. The genetic risk for type 2 diabetes was stratified into low (lowest PRS tertile), intermediate (middle PRS tertile), and high (highest PRS tertile) categories based on the standard PRS. Cox adjusted for age, sex, British descent, Townsend deprivation index (TDI), assessment centers, family history of diabetes, hypertension, body mass index, waist circumference, smoking status, alcohol intake frequency, left ventricular longitudinal strain global, left ventricular ejection fraction and left atrial ejection fraction. CI, confidence interval; HR, hazard ratio

Participants were classified into low, intermediate, and high genetic risk groups according to their standard PRS level. It was observed that among those with normal LV size, the highest risk for type 2 diabetes was found in the high genetic risk group. When participants with normal LV size and low genetic risk were treated as the reference group, it was found that those with small LV size and high genetic risk had the highest risk for type 2 diabetes (adjusted HR, 6.55; 95% CI, 3.65–11.77) (Fig. 3).

**Fig. 4:**
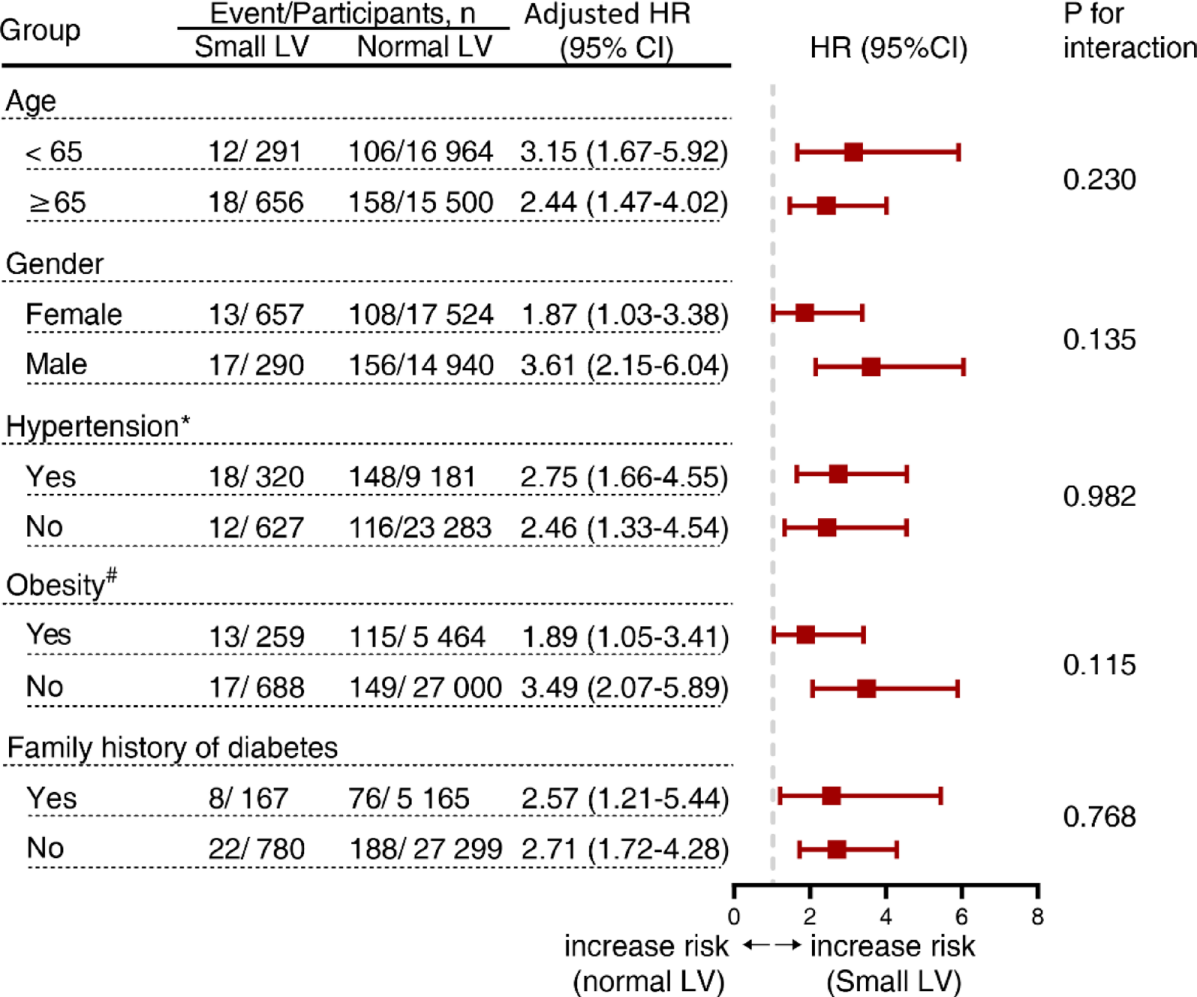
Subgroup analysis and interaction of subgroup variables on the association between small left ventricle and incident type 2 diabetes. *Hypertension include the history of hypertension and anti-hypertensive medication uses. #Obesity includes body mass index ≥ 30 kg/m2 and abdominal obesity (waist circumference ≥ 105 cm for female and ≥ 110 cm for male). Cox adjusted for age, sex, British descent, Townsend deprivation index (TDI), assessment centers, family history of diabetes, hypertension, body mass index, waist circumference, smoking status, alcohol intake frequency, left ventricular longitudinal strain global, left ventricular ejection fraction, left atrial ejection fraction and polygenic risk scores for type 2 diabetes

Further subgroup analysis was conducted, stratifying participants according to age, sex, hypertension, obesity, and family history of diabetes. When compared to those with normal LV size, an increased risk for type 2 diabetes was consistently observed in participants with small LV size across various subgroups, without significant differences being noted (Fig. 4).

### Correlation of left ventricular size with clinical characteristics and cardiac function

The evaluation of the relationship between LV size, represented as a continuous variable (iLVEDV), and SBP, BMI, and cardiac function has been carried out (Figure S1). The results revealed that iLVEDV exhibited a positive correlation with SBP, LV stroke volume, LV cardiac output, LV longitudinal strain, and indexed LV mass (iLVmass), while showing a negative correlation with BMI, heart rate, and LVEF. A non-linear relationship was observed between iLVEDV and these clinical parameters.

### Association between small left ventricular size and frailty

The odds ratio (OR) of FI for small LV size was estimated using a multiple-variables adjusted logistic regression model. With the increase in FI, a gradual increase in the OR for small LV size was observed (Figure S2). In males, significant association between frailty status and small LV size was found (adjusted OR, 1.67; 95% CI, 1.05–2.65, *P* = 0.025), whereas such association was not statistically significant in females (adjusted OR, 1.35; 95% CI, 0.99–1.85, *P* = 0.061).

### Sensitivity analyses

Firstly, the association of LV size with type 2 diabetes was re-evaluated using a competing risk model, in which all-cause death was treated as the competitive events. Through this method, small LV size exhibited an adjusted subdistribution hazard ratio of 2.12 (95% CI, 1.46–3.08) for type 2 diabetes compared to normal LV size (Table S3). Secondly, the Cox proportional hazards model was re-analyzed with age as the time-scale, revealing an adjusted HR of 2.14 (95% CI, 1.42–3.21) (Table S4). Thirdly, a total of 31,802 participants were included for analysis after excluding those with previously recorded abnormal HbA1c results (Table S5). Among these participants, 776 were classified into small LV size and exhibited a 1.69 times higher risk for type 2 diabetes compared to those with normal LV size (adjusted HR, 2.69; 95% CI, 1.56–4.63). Fourthly, those with missing data on covariates were excluded (Table S6). In this complete dataset, small LV size was still associated with an increased risk for type 2 diabetes (adjusted HR, 2.95; 95% CI, 1.85–4.73). Finally, Cox Model 3 in Table 1 was further adjusted for the LV mass-to-volume ratio, revealing an adjusted hazard ratio of 1.63 (95% CI, 1.06–2.51, *P* = 0.027).  After further adjusting for baseline dyslipidemia in addition to all covariates included in Model 3, small LV size remained significantly associated with an increased risk of incident type 2 diabetes (adjusted HR, 2.26; 95% CI, 1.49–3.43). Similarly, for each 10 mL/m² decrease in iLVEDV, the risk of developing type 2 diabetes increased by 33% (adjusted HR, 1.33; 95% CI, 1.17–1.50; *p* < 0.001). The Firth penalized Cox regression and reduced-variable sensitivity analyses were materially unchanged from our main analyses, supporting the robustness of our conclusions despite the smaller event count in the small LV size group (the detailed results in the Table S9 and Table S10).

## Discussion

In the present study, we have several findings: (1) The size of the LV, as measured by CMR, exhibited a positive correlation with LV mass in the UKB cohort. Participants with smaller LV size demonstrated lower LV mass compared to their counterparts; (2) Small LV size is associated with increased risk for incident type 2 diabetes, irrespective of background genetic risks, age, sex, and other metabolic factors such as hypertension and obesity; (3) Frail status showed a notable association with small LV size.

The direct correlation between diabetes and myocardial remodeling was first reported in the 1950 s and has since been extensively studied [36]. Diabetic cardiomyopathy, also termed diabetic myocardial disorder, is characterized by structural and functional abnormalities of the myocardium in patients with diabetes in the absence of coronary artery disease, hypertension, or obesity. According to the recent European Heart Failure Association consensus statement [37]. It is not difficult to comprehend the chronological progression of diabetic cardiomyopathy. Hyperglycemia, as a pivotal component of metabolic disorders, disrupts the balance in energy metabolism, thereby fostering cardiac hypertrophy and fibrosis, ultimately culminating in LV remodeling and dysfunction [38]. Notably, recent research findings prompt us to reevaluate the sequence of myocardial structural abnormalities and diabetes. In a cohort of 4176 primary hypertension patients without diabetes and cardiovascular complications, the presence of LV hypertrophy (defined as iLVmass ≥ 51 g/m^2^) was associated with a 63% increase in the likelihood of incident diabetes [7]. This association between LV structural changes and incident diabetes was subsequently confirmed in the Copenhagen City Heart Study cohort, where individuals from the general population exhibiting LV concentric remodeling or hypertrophy on echocardiography demonstrated a higher risk of developing diabetes [8].

In our findings from CMR, individuals with small LV size exhibit a higher LV mass to volume ratio, a conceptually analogous metric to the echocardiogram-derived relative wall thickness [39]. Although LV wall thickness is available in the UK Biobank CMR dataset, we used the LV mass-to-volume ratio (MVR) as a proxy for relative wall thickness for two reasons. First, in CMR, MVR is a well-validated index of concentric remodeling that incorporates both wall thickness and chamber size, and is conceptually analogous to the echocardiographic relative wall thickness [40, 41]. Second, compared with single-slice wall thickness measurements, MVR is less susceptible to measurement variability due to slice positioning or endocardial/epicardial contouring, thereby providing a more robust characterization of LV geometry in large-scale population studies [42–45]. This ratio reflects the extent of LV concentric change and indicates that LV concentric remodeling is the feature of small LV size, thereby connecting our results with previous findings. The link between small LV size and diabetes remained unaffected by age and other metabolic risk factors—such as hypertension and obesity—that are known to be closely associated with both LV structural changes and diabetes, thus mitigating potential confounding effects [46, 47]. However, participants with a small LV size in our study exhibit a corresponding low LV mass, distinguishing our findings from previous studies where LV structural changes preceding diabetes involved increased LV mass and consequently reduced LV size [7–9]. On the contrary, our results share similarity with that of the community-based Atherosclerosis Risk In the Community (ARIC) study, which found individuals with pre-diabetic status have a smaller LV size while the wall thickness and LV mass are comparable to those without glucose abnormalities [11]. Notably, despite adjustments for relative wall thickness (LV mass to volume ratio), small LV size remains significantly associated with incident type 2 diabetes, suggesting its independent role as a predictor for developing diabetes.

Several factors may potentially account for our findings. Firstly, LV size tends to decrease with increasing age in healthy individuals, and small LV size may serve as a predictor for metabolic disorders associated with aging. A recent study recruited a healthy population ranging in age from 19 to over 50 years old to undergo CMR examinations. The study showed a gradual decrease in both LV size and mass as individuals aged [48]. This negative correlation between LV size and age in the healthy population has been consistently supported by previous studies [49]. As aging progresses, cellular senescence, inflammation activation, and mitochondrial dysfunction occur, all of which contribute to the progression of glucose dysregulation^25^. Furthermore, findings from ARIC studies corroborated this explanation, which found a significant reduced LV size but normal LV mass among individuals with pre-diabetic condition compared to those without diabetes, even after adjusting for other metabolic risks such as blood pressure, BMI and cholesterol [11, 50]. It is worth noting that the average age of the participants in ARIC study was 75 years. In contrast, increased LV mass and LV hypertrophy are frequently observed among adolescents, young adults and middle-aged individuals with pre-diabetic status [51, 52].

The second possible explanation refers to the association between low birth weight and diabetes. Findings from a large-scale meta-analysis revealed a J-shaped relationship between birth weight and the risk of type 2 diabetes, hypertension and cardiovascular diseases with an increased risk discerned among individuals with low birth weight [53]. A cross-sectional population-based study involving middle-aged participants revealed that low birth weight is significantly associated with diabetes and obesity, underscoring the long-lasting impact of birth weight on metabolic health [54]. LV size is notably influenced by birth weight. According to the real-world data, there exists a Linear correlation between low birth weight and reduced LV size in adulthood, with robust correlation coefficients of 0.70 (95% CI, 0.51–0.90, *P* < 0.001) [55]. This correlation, indicating small LV size in individuals with low birth weight, is further supported by causal estimates derived from Mendelian randomization analysis [56]. Based on our results, those with small LV size have a lower birth weight and higher percentages of metabolic disorders compared to their counterparts. Given the life-long impact of birth weight on metabolism, small LV size may present as an indicator susceptibility to diabetes. Further exploration into the interplay between low birth weight and small LV size is warranted.

Small LV size is also a characteristics of small heart syndrome, a condition primarily reported by Japanese scholars [57, 58]. Diagnosis of small heart syndrome relies on chest radiography (with a cardiothoracic ratio ≤ 42%), with chronic fatigue being the primary clinical presentation observed in the affected individuals [57]. This syndrome is also regarded as low output syndrome, as the primary issue within this population is low cardiac output [58].This reduction in cardiac function leads to inadequate tissue perfusion, resulting in fatigue symptoms. Although there’s a gap in prospective data linking fatigue directly to the onset of diabetes, emerging evidence suggests a correlation between frailty—a more comprehensive concept encompassing fatigue [30]—and the development of type 2 diabetes [59, 60]. Notably, a significant negative linear relationship exists between the frailty index and LV size, and participants identified as frail exhibit elevated odds of having small LV size in this study. A previous study involving community-dwelling old women without known diabetes, found that frailty was associated with impaired responses to glucose loading, indicating compromised glucose-insulin regulation [61]. Moreover, the levels of circulating antioxidant decreased in frail elderly patients, which exacerbates oxidative stress and promotes the progression of aging-related disease [62].

Based on current evidence, establishing a causal link between small LV size and T2D proves challenging. In practice, universal CMR screening is unlikely to be cost-effective; however, opportunistic echocardiography-based assessment during routine cardiovascular evaluation is feasible and scalable [63]. Pending formal calibration studies to translate CMR thresholds to echocardiographic indices, a practical approach is to flag individuals in the lowest age- and sex-specific decile of indexed LV end-diastolic volume (or LV end-diastolic diameter) for intensified diabetes surveillance [64, 65]. Risk-enriched targeting is also plausible: people with a history of low birth weight—which tracks with smaller adult LV size and higher metabolic risk—could be prioritized for imaging and lifestyle counselling [55, 66]. Once identified, at-risk individuals can be engaged through telemedicine-enabled programs (remote glucose checks, activity coaching, and nutrition support), an approach that has shown improvements in metabolic profiles and aligns with our observation that small LV size may capture a phenotype responsive to lifestyle modification [67, 68]. Taken together, a stepwise strategy—risk enrichment (e.g., low birth weight, frailty, or cardiometabolic clustering), opportunistic echocardiographic screening for small LV size, selective confirmatory CMR when indicated, and telemedicine-enabled lifestyle interventions—offers a feasible pathway to translate our results while minimizing costs and expanding access [69].

Several limitations warrant consideration. Firstly, participants who underwent CMR assessment in the UK Biobank did not always have concurrent laboratory measurements, and the availability of HbA1c or glucose data on the same day as CMR imaging was limited. Consequently, we could not fully examine correlations between glycaemic indices and LV size, which may introduce residual confounding. As a sensitivity analysis, we excluded individuals with an HbA1c value greater than 5.7%, indicative of a prediabetic condition. Secondly, the study population primarily comprised British individuals of white ethnicity, which may Limit the generalizability of our findings to other ethnic groups and requires validation in more diverse populations. Thirdly, the study only included middle-aged and older adults, resulting in a Limited age range. Further research is warranted to determine whether the association between small LV size and type 2 diabetes risk is also present in younger adults. Fourthly, although events per variable is defined with respect to the total number of events in the model [29], sparse events in the exposure category may still induce sparse-data bias and wide CIs; we therefore applied Firth penalized Cox regression and reduced-variable sensitivity analyses, which produced consistent results.

## Conclusions

Individuals with small LV size, as measured by CMR, demonstrate reduced iLVEDV and iLVmass compared to their counterparts. Small LV size independently predict the incidence of type 2 diabetes, irrespective of age, sex, hypertension, obesity, and LV concentric remodeling. Additional research is needed to further investigate the association between small LV size and type 2 diabetes.

## Supplementary Information

Supplementary Material 1

## Data Availability

All data used in this work can be acquired from the UK Biobank (https://www.ukbiobank.ac.uk/), subject to obtaining official permission from UK Biobank.

## Abbreviations

CMR: Cardiovascular magnetic resonance
FI: Frailty index
iLVEDV: Indexed left ventricular end diastolic volume
PRS: Polygenic risk scores
UKB: UK Biobank
