# Supplementary material for: Small left ventricular size as a predictor for incident type 2 diabetes: insights from the UK biobank cardiovascular magnetic resonance substudy
Source: Diabetol Metab Syndr. 2025 Oct 3;17:375. doi: 10.1186/s13098-025-01939-7 (PMC12495618; doi:10.1186/s13098-025-01939-7)
Supplement: Supplementary file 1 — Supplementary Material 1 [file 13098_2025_1939_MOESM1_ESM.docx]

Small left ventricular size as a predictor for incident type 2 diabetes: insights from the UK Biobank Cardiovascular Magnetic Resonance Substudy

Table of Content

[Table S1. Variables and outcome definition 2](#_Toc166757308)

[Table S2. Variables used for the calculation of frailty index 3](#_Toc166757309)

[Table S3. Association of left ventricle size and incident type 2 diabetes using competing risk model (n=35 422) 5](#_Toc166757310)

[Table S4. Association of small left ventricle size and incident type 2 diabetes using age as time scale (n=35 422) 6](#_Toc166757311)

[Table S5. Association of small left ventricle size and incident type 2 diabetes after excluding participants with prior HbA1c level>5.7% (n=31 802) 7](#_Toc166757312)

[Table S6. Association of small left ventricle size and incident type 2 diabetes using complete dataset (n=27 251) 8](#_Toc166757313)

Table S7 Multicollinearity Diagnostics for Model Covariates ........................................... 9

Table S8. Assessment of normality for study variables.....................................................10

Table S9. Association of left ventricle size and incident type 2 diabetes using Firth’s penalized likelihood Cox regression..................................................................................11

Table S10. Association of left ventricle size and incident type 2 diabetes using Cox proportional hazard model (model 5 and model6).............................................................12

[Figure S1. Correlation of indexed left ventricular end diastolic volume with anthropometric and cardiac function (n=35 422) 1](#_Toc166757314)3

[Figure S2. The sex-specific distribution and association of frailty with small left ventricular size (n=24 799) 14](#_Toc166757315)

Table S1. Variables and outcome definition

| **Variable** | **UKB data-field link^#^** |
| --- | --- |
| Age | p21003 |
| Sex | p31 |
| Ethnic background | p21000 |
| Smoking status | p20116 |
| Alcohol intake frequency | p1558 |
| TDI | p22189 |
| SBP | p4080 |
| DBP | p4079 |
| Heart rate | p22426 |
| Weight | p21002 |
| Height | p50 |
| BMI | p21001 |
| Waist circumference | p48 |
| Body surface area | p22427 |
| Family history of diabetes | p20107, p20110, p20111 |
| Cardiomyopathy | p131338 |
| Diabetes | p130706, p130708, p130710, p130712, p130714 |
| Hypertension | p131286 |
| Medication for diabetes, hypertension | p6177, p6153 |
| Glycated hemoglobin (HbA1c) | p30750 |
| LVEDV | p24100 |
| LVESV | p24101 |
| LV myocardial mass | p24105 |
| LV cardiac output | p24104 |
| LV stroke volume | p24102 |
| LV longitudinal strain global | p24181 |
| LVEF | p24103 |
| LA maximum volume | p24110 |
| LA minimum volume | p24111 |
| LAEF | p24113 |
| PRS for T2D | p26285 |
| **Outcome** | **ICD-10 code** |
| Incident T2D | E11.0, E11.1, E11.2, E11.3, E11.4, E11.5, E11.6, E11.7, E11.8, E11.9 |

^#^Note: Table S1 provides the full list and definitions of variables available in the UKB and used in various stages of this study.

Link can be tracked through the website: <https://biobank.ctsu.ox.ac.uk/crystal/browse.cgi?id=-2&cd=search>

BMI, body mass index; DBP, diastolic blood pressure; LA, left atrial; LV, left ventricular; LVEDV, left ventricular end diastolic volume; LVEF, left ventricular ejection fraction; LVESV, left ventricular end systolic volume; PRS, polygenic risk scores; SBP, systolic blood pressure; TDI, Townsend deprivation index; T2D, type 2 diabetes

Table S2. Variables used for the calculation of frailty index

| **Variable** | **UKB**  **data-field link^#^** | **Score calculation** |
| --- | --- | --- |
| **Sensory** |  |  |
| Glaucoma * | p6148 | Categorized 0/1 |
| Cataracts * | p6148 | Categorized 0/1 |
| Hearing difficulty | p2247 | Categorized 0/1  (combined yes/deaf groups as 1) |
| **Cranial** |  |  |
| Migraine * | p20002 | Categorized 0/1 |
| Dental problems | p6149 | Categorized 0/1 for none vs. any |
| **Mental wellbeing** |  |  |
| Self-rated health | p2178 | 0 – excellent; 0.25 – good;  0.5 - fair, 1 – poor |
| Fatigue: frequency of tiredness / lethargy in last two weeks | p2080 | 0, 0.25, 0.5, 1, respectively |
| Sleep: experience of sleeplessness/insomnia | p1200 | Categorised 0, 0.5, 1, respectively |
| Depressed feelings: frequency in last two weeks | p2050 | 0 – not at all, 0.5 – several days, 0.75 -- more than half, 1 – nearly every day |
| Self-described nervous personality | p1970 | Categorised 0/1 |
| Severe anxiety/ panic attacks * | p20002 | Categorised 0/1 |
| Common to feel loneliness | p2020 | Categorised 0/1 |
| Sense of misery (ever/never) | p1930 | Categorised 0/1 |
| **Infirmity** |  |  |
| Infirmity: long-standing illness or disability | p2188 | Categorised 0/1 |
| Falls in last year | p2296 | 0, 0.5, 1, respectively |
| Fractures/broken bones in last five years | p2463 | Categorised 0/1 |
| **Cardiometabolic** |  |  |
| Diabetes * | p20002 | Categorised 0/1 |
| Myocardial infarction * | p20002 | Categorised 0/1 |
| Angina * | p20002 | Categorised 0/1 |
| Stroke * | p20002 | Categorised 0/1 |
| High blood pressure * | p20002 | Categorised 0/1 |
| Hypothyroidism * | p20002 | Categorised 0/1 |
| Deep-vein thrombosis * | p6152 | Categorised 0/1 |
| High cholesterol * | p20002 | Categorised 0/1 |
| **Respiratory** |  |  |
| Breathing: wheeze in last year | p2316 | Categorised 0/1 |
| Pneumonia * | p20002 | Categorised 0/1 |
| Chronic bronchitis/emphysema * | p20002 | Categorised 0/1 |
| Asthma * | p20002 | Categorised 0/1 |
| **Gastrointestinal** |  |  |
| Gastric reflux * | p20002 | Categorised 0/1 |
| Hiatus hernia * | p20002 | Categorised 0/1 |
| Gall stones * | p20002 | Categorised 0/1 |
| Diverticulitis * | p20002 | Categorised 0/1 |
| **Musculoskeletal** |  |  |
| Rheumatoid arthritis * | p20002 | Categorised 0/1 |
| Osteoarthritis * | p20002 | Categorised 0/1 |
| Gout * | p20002 | Categorised 0/1 |
| Osteoporosis * | p20002 | Categorised 0/1 |
| **Immunological** |  |  |
| Hayfever, allergic rhinitis or eczema * | p6152 | Categorised 0/1 |
| Psoriasis * | p20002 | Categorised 0/1 |
| **Cancer** |  |  |
| Any cancer diagnosis * | p2453 | Categorised 0/1 |
| Multiple cancers diagnosed (number reported) | p134 | 0 - no cancer or single cancer, 1 - multiple cancers |
| **Pain** |  |  |
| Chest pain | p2335 | Categorised 0/1 |
| Head and/or neck pain | p3799 p3404 | Categorised 0/1 |
| Back pain | p3571 | Categorised 0/1 |
| Stomach/abdominal pain | p3741 | Categorised 0/1 |
| Hip pain | p3414 | Categorised 0/1 |
| Knee pain | p3773 | Categorised 0/1 |
| Whole-body pain | p2956 | Categorised 0/1 |
| Facial pain | p4067 | Categorised 0/1 |
| Sciatica * | p20002 | Categorised 0/1 |

#Link can be tracked through the website: <https://biobank.ctsu.ox.ac.uk/crystal/browse.cgi?id=-2&cd=search>

* Participants reported medically diagnosed conditions related to these variables

Table S3. Association of left ventricle size and incident type 2 diabetes using competing risk model (n=35 422)

|  | **Incident event, No.** | **Competing event, No.** | **Participants No.** | **Subdistribution Hazard Ratio (95% CI)** | | |
| --- | --- | --- | --- | --- | --- | --- |
|  |  |  |  | **Model 1** | **Model 2** | **Model 3** |
| **Continuous variable** | |  |  |  |  |  |
| iLVEDV per 10mL/m^2^ decrease | 304 | 607 | 35 422 | 1.26 (1.15-1.39)  P<0.001 | 1.37 (1.23-1.52)  P<0.001 | 1.35 (1.22-1.51)  P<0.001 |
| **Categorical variable*** | |  |  |  |  |  |
| Normal LV size | 264 | 542 | 32 464 | Reference | Reference | Reference |
| Small LV size | 30 | 20 | 947 | 2.32 (1.62-3.31)  P<0.001 | 2.21 (1.52-3.21)  P<0.001 | 2.12 (1.46-3.08)  P<0.001 |
| Large LV size | 10 | 45 | 2 011 | 0.88 (0.68-1.15)  P=0.44 | 0.91 (0.70-1.20)  P=0.57 | 0.91 (0.70-1.20)  P=0.58 |

Model 1: age, sex, British descent, Townsend deprivation index (TDI), assessment centers, family history of diabetes hypertension, body mass index, waist circumference, smoking status and alcohol intake frequency

Model 2: Model 1 plus, left ventricular mass, left ventricular longitudinal strain global, left ventricular ejection fraction and left atrial ejection fraction

Model 3: Model 2 plus polygenic risk scores for type 2 diabetes

*Classification of left ventricle size was based on the European Association of Cardiovascular Imaging expert consensus on cardiovascular magnetic resonance normal values of cardiac chamber size

Table S4. Association of small left ventricle size and incident type 2 diabetes using age as time scale (n=35 422)

|  | **Events**  **No.** | **Participants No.** | **Hazard ratio (95% CI)** | | |
| --- | --- | --- | --- | --- | --- |
|  |  |  | **Model 1** | **Model 2** | **Model 3** |
| Continuous variable |  |  |  |  |  |
| iLVEDV  per 10mL/m^2^ decrease | 304 | 35 422 | 1.16(1.06-1.27)  P=0.001 | 1.30 (1.16-1.46)  P<0.001 | 1.27 (1.14-1.42)  P<0.001 |
| Categorical variable* |  |  |  |  |  |
| Normal LV size | 264 | 32 464 | Reference | Reference | Reference |
| Small LV size | 30 | 947 | 2.61 (1.79-3.81)  P<0.001 | 2.35 (1.56-3.53)  P<0.001 | 2.14 (1.42-3.21)  P<0.001 |
| Large LV size | 10 | 2 011 | 0.72 (0.38-1.35)  P=0.300 | 0.69 (0.35-1.36)  P=0.280 | 0.69 (0.35-1.36)  P=0.280 |

Model 1: age, sex, British descent, Townsend deprivation index (TDI), assessment centers, family history of diabetes hypertension, body mass index, waist circumference, smoking status and alcohol intake frequency

Model 2: Model 1 plus, left ventricular mass, left ventricular longitudinal strain global, left ventricular ejection fraction and left atrial ejection fraction

Model 3: Model 2 plus polygenic risk scores for type 2 diabetes

*Classification of left ventricle size was based on the European Association of Cardiovascular Imaging expert consensus on cardiovascular magnetic resonance normal values of cardiac chamber size

Table S5. Association of small left ventricle size and incident type 2 diabetes after excluding participants with prior HbA1c level>5.7% (n=31 802)

|  | **Events**  **No.** | **Participants^#^ No.** | **Hazard ratio (95% CI)** | | |
| --- | --- | --- | --- | --- | --- |
|  |  |  | **Model 1** | **Model 2** | **Model 3** |
| Continuous variable |  |  |  |  |  |
| iLVEDV  per 10mL/m^2^ decrease | 170 | 31 802 | 1.27 (1.12-1.44)  P<0.001 | 1.37 (1.16-1.60)  P<0.001 | 1.36 (1.16-1.59)  P<0.001 |
| Categorical variable* |  |  |  |  |  |
| Normal LV size | 145 | 29 164 | Reference | Reference | Reference |
| Small LV size | 18 | 776 | 2.79 (1.68-4.62)  P<0.001 | 2.82 (1.64-4.84)  P<0.001 | 2.69 (1.56-4.63)  P<0.001 |
| Large LV size | 7 | 1 862 | 0.93 (0.44-2.00)  P=0.885 | 0.95 (0.42-2.14)  P=0.896 | 0.95 (0.42-2.15)  P=0.909 |

^#^ Excluding participants who have ever had a laboratory test indicating glycated hemoglobin (HbA1c) levels >5.7% (indicative of pre-diabetic and diabetic status) (n=3,620)

more than 10% missing data on variables used to calculate the frailty index (n=9 814)

Model 1: age, sex, British descent, Townsend deprivation index (TDI), assessment centers, family history of diabetes hypertension, body mass index, waist circumference, smoking status and alcohol intake frequency

Model 2: Model 1 plus, left ventricular mass, left ventricular longitudinal strain global, left ventricular ejection fraction and left atrial ejection fraction

Model 3: Model 2 plus polygenic risk scores for type 2 diabetes

*Classification of left ventricle size was based on the European Association of Cardiovascular Imaging expert consensus on cardiovascular magnetic resonance normal values of cardiac chamber size

Table S6. Association of small left ventricle size and incident type 2 diabetes using complete dataset (n=27 251)

|  | **Events**  **No.** | **Participants^#^ No.** | **Hazard ratio (95% CI)** | | |
| --- | --- | --- | --- | --- | --- |
|  |  |  | **Model 1** | **Model 2** | **Model 3** |
| Continuous variable |  |  |  |  |  |
| iLVEDV  per 10mL/m^2^ decrease | 218 | 27 251 | 1.27 (1.13-1.42)  P<0.001 | 1.36 (1.18-1.57)  P<0.001 | 1.35 (1.17-1.55)  P<0.001 |
| Categorical variable* |  |  |  |  |  |
| Normal LV size | 188 | 25 022 | Reference | Reference | Reference |
| Small LV size | 24 | 661 | 3.10 (2.00-4.79)  P<0.001 | 3.08 (1.92-4.92)  P<0.001 | 2.95 (1.85-4.73)  P<0.001 |
| Large LV size | 6 | 1 568 | 0.61 (0.27-1.38)  P=0.237 | 0.58 (0.25-1.36)  P=0.208 | 0.57 (0.24-1.34)  P=0.199 |

^#^Excluding participants with missing data on variables used for adjusting Cox model (n=8 171)

Model 1: age, sex, British descent, Townsend deprivation index (TDI), assessment centers, family history of diabetes hypertension, body mass index, waist circumference, smoking status and alcohol intake frequency

Model 2: Model 1 plus, left ventricular mass, left ventricular longitudinal strain global, left ventricular ejection fraction and left atrial ejection fraction

Model 3: Model 2 plus polygenic risk scores for type 2 diabetes

*Classification of left ventricle size was based on the European Association of Cardiovascular Imaging expert consensus on cardiovascular magnetic resonance normal values of cardiac chamber size

Table S7 Multicollinearity Diagnostics for Model Covariates

| Variable | Tolerance | VIF |
| --- | --- | --- |
| Sex | 0.439 | 2.278 |
| Age | 0.861 | 1.161 |
| British descent | 0.972 | 1.028 |
| Family history of diabetes | 0.974 | 1.027 |
| Hypertension | 0.884 | 1.131 |
| Assessment centers | 0.959 | 1.042 |
| Smoking status | 0.949 | 1.053 |
| Alcohol_intake_frequency | 0.929 | 1.077 |
| Waist circumference | 0.642 | 1.558 |
| Townsend deprivation index | 0.952 | 1.050 |
| Left ventricular longitudinal strain global | 0.740 | 1.352 |
| Left ventricular ejection fraction | 0.741 | 1.350 |
| Left ventricular mass | 0.374 | 2.673 |
| Left atrial ejection fraction | 0.863 | 1.158 |
| Polygenic risk scores for type 2 diabetes | 0.972 | 1.029 |
| Body mass index | 0.270 | 3.709 |

Note: All Tolerance values >0.2 and VIF values <5, suggesting no significant multicollinearity among model covariates.

Table S8. Assessment of normality for study variables using the Kolmogorov–Smirnov test with Lilliefors significance correction

| Variable | K–S Statistic | df | p-value | Normality* |
| --- | --- | --- | --- | --- |
| Age, years | 0.058 | 21329 | <0.001 | No |
| Townsend deprivation index, TDI | 0.114 | 21329 | <0.001 | No |
| SBP, mmHg | 0.039 | 21329 | <0.001 | No |
| DBP, mmHg | 0.035 | 21329 | <0.001 | No |
| Heart rate, bpm | 0.065 | 21329 | <0.001 | No |
| BMI, kg/m2 | 0.062 | 21329 | <0.001 | No |
| Waist circumference, cm | 0.039 | 21329 | <0.001 | No |
| Birth weight, kg | 0.091 | 21329 | <0.001 | No |
| Fragility index | 0.081 | 21329 | <0.001 | No |
| PRS for type 2 diabetes | 0.005 | 21329 | 0.145 | Yes |
| iLVEDV, mL/m2 | 0.044 | 21329 | <0.001 | No |
| iLVESV, mL/m2 | 0.060 | 21329 | <0.001 | No |
| LV cardiac output, L/min | 0.053 | 21329 | <0.001 | No |
| LV stroke volume, mL | 0.047 | 21329 | <0.001 | No |
| LVEF, % | 0.019 | 21329 | <0.001 | No |
| LV longitudinal strain global, % | 0.023 | 21329 | <0.001 | No |
| LA maximum volume, mL | 0.050 | 21329 | <0.001 | No |
| LA minimum volume, mL | 0.084 | 21329 | <0.001 | No |
| LAEF, % | 0.048 | 21329 | <0.001 | No |
| Indexed LV myocardial mass, g/m2 | 0.062 | 21329 | <0.001 | No |

*Normality defined as p > 0.05.

Table S9. Association of left ventricle size and incident type 2 diabetes using Firth’s penalized likelihood Cox regression

|  | Events  No. | Participants No. | Hazard ratio (95% CI) | | |
| --- | --- | --- | --- | --- | --- |
|  |  |  | Model 1 | Model 2 | Model 3 Model4 |
| Continuous variable |  |  |  |  |  |
| iLVEDV  per 10mL/m2 decrease | 304 | 35 422 | 1.25 (1.14-1.38)  P<0.001 | 1.35 (1.20-1.53)  P<0.001 | 1.34 (1.19-1.51) 1.33(1.18-1.50  P<0.001 P<0.001 |
| Categorical variable* |  |  |  |  |  |
| Normal LV size | 264 | 32 464 | Reference | Reference | Reference Reference |
| Small LV size | 30 | 947 | 2.47(1.65-3.57)  P<0.001 | 2.45 (1.59-3.64)  P<0.001 | 2.37 (1.54-3.52) 2.30(1.50- 3.43)  P<0.001 P<0.001 |
| Large LV size | 10 | 2 011 | 0.84(0.42-1.48)  P=0.574 | 0.83 (0.41-1.53)  P=0.577 | 0.85 (0.41-1.56) 0.86( 0.42-1.58)  P=0.609 P=0.642 |

Model 1: age, sex, British descent, Townsend deprivation index (TDI), assessment centers, family history of diabetes, hypertension, body mass index, waist circumference, smoking status and alcohol intake frequency

Model 2: Model 1 plus, left ventricular mass, left ventricular longitudinal strain global, left ventricular ejection fraction and left atrial ejection fraction

Model 3: Model 2 plus polygenic risk scores for type 2 diabetes

Model 4: Model 3 plus Dyslipidemia

*Classification of left ventricle size was based on the European Association of Cardiovascular Imaging expert consensus on cardiovascular magnetic resonance normal values of cardiac chamber size

Table S10. Association of left ventricle size and incident type 2 diabetes using Cox proportional hazard model (model 5 and model6)

|  | Events No. | Participants No. | Hazard ratio (95% CI) - Model 5 | Hazard ratio (95% CI)- Model 6 |  |
| --- | --- | --- | --- | --- | --- |
| iLVEDV per 10mL/m2 decrease | 304 | 35422 | 1.41 (1.28-1.54) P<0.001 | 1.31 (1.20-1.44) P<0.001 |  |
| Normal LV size | 264 | 32464 | Reference | Reference |  |
| Small LV size | 30 | 947 | 4.49 (3.07-6.55) P<0.001 | 3.40 (2.33-4.98) P<0.001 |  |
| Large LV size | 10 | 2011 | 0.57 (0.30-1.07) P=0.078 | 0.65 (0.35-1.22) P=0.180 |  |

NOTE:

Model 5: Univariable Cox proportional hazards regression.

Model 6:adjusted for age, family history of diabetes, polygenic risk scores for type 2 diabetes.

*Classification of left ventricle size was based on the European Association of Cardiovascular Imaging expert consensus on cardiovascular magnetic resonance normal values of cardiac chamber size


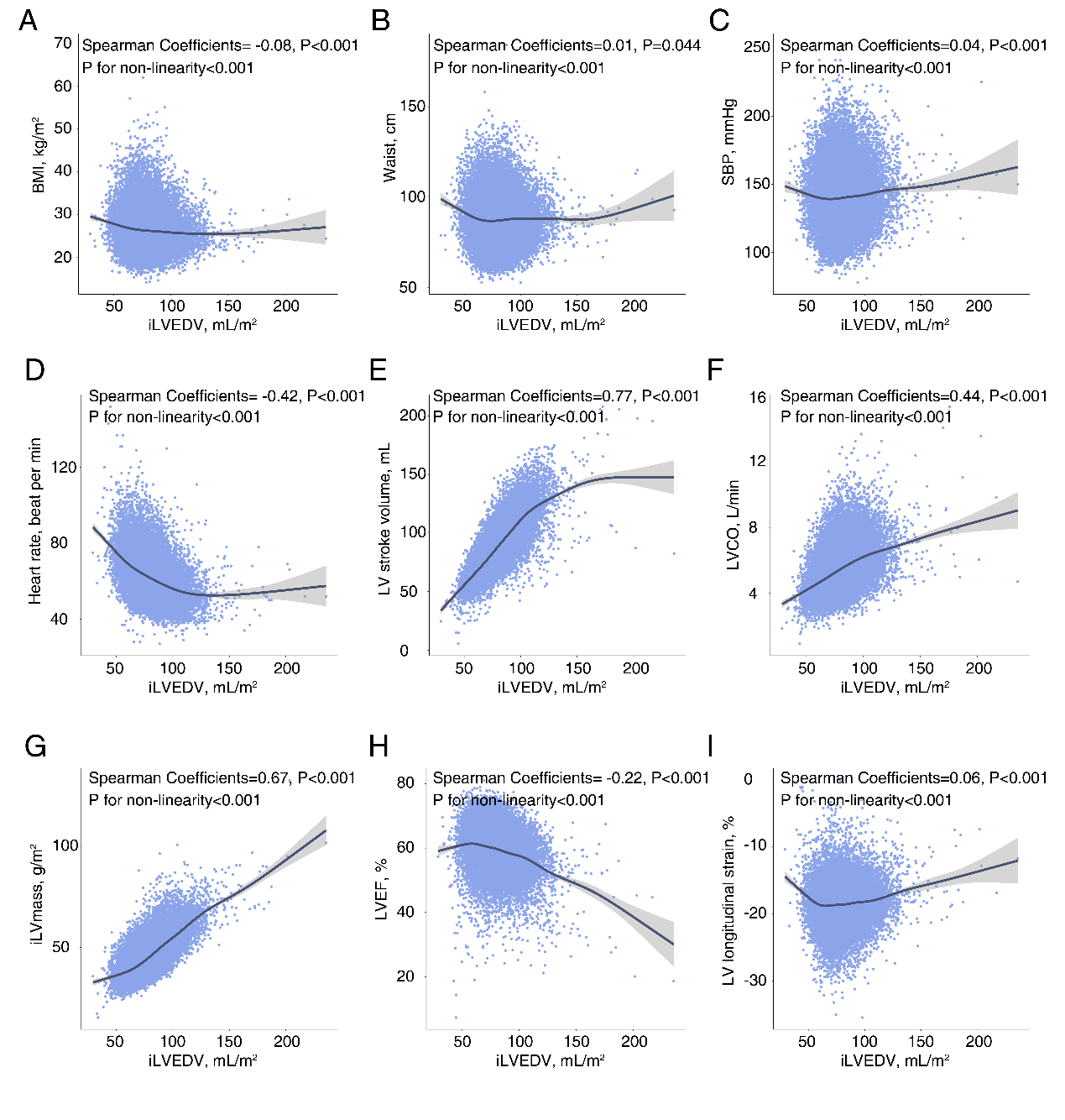


Figure S1. Correlation of indexed left ventricular end diastolic volume with anthropometric and cardiac function (n=35 422)

A. body mass index (BMI); B. waist circumference; C. systolic blood pressure (SBP); D. heart rate; E. left ventricle stroke volume; F. left ventricle cardiac output (LVCO); G. indexed left ventricular mass (iLVmass); H. left ventricular ejection fraction (LVEF); I. left ventricular longitudinal strain


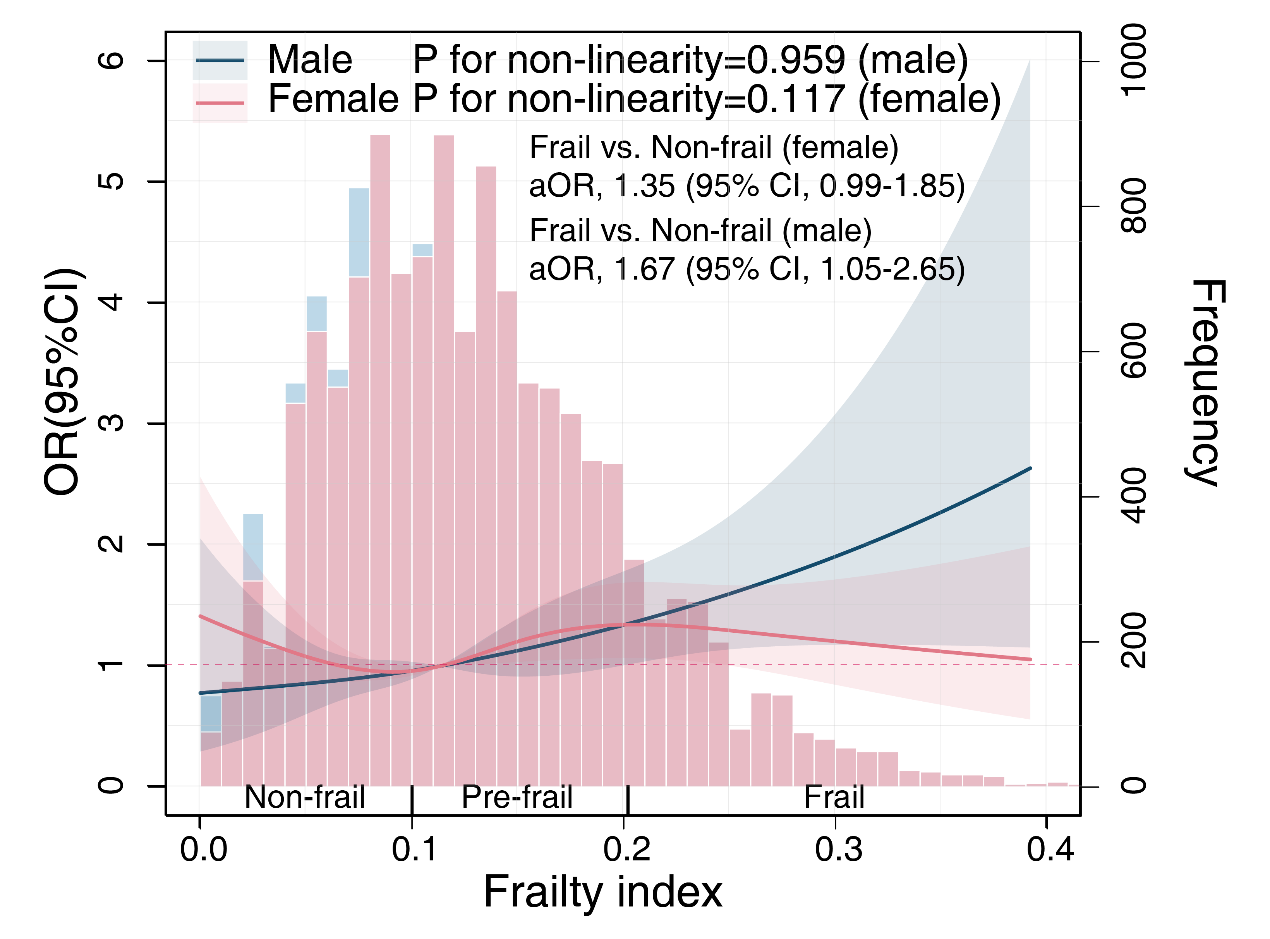


Figure S2. The sex-specific distribution and association of frailty with small left ventricular size (n=24 799)

Excluding participants with more than 10% missing data on variables used to calculate the frailty index (n=10 623)

Non-linear relationship between frailty index and small left ventricular size using adjusted logistic regression model stratified by sex

Logistic regression model adjusted for age, British descent, Townsend deprivation index (TDI), assessment centers, family history of diabetes, hypertension, body mass index, waist circumference, smoking status, alcohol intake frequency, left ventricular longitudinal strain global, left ventricular ejection fraction, left atrial ejection fraction and polygenic risk scores for type 2 diabetes

CI, confidence interval; OR, odds ratio
